# Current Status of Autophagy Enhancers in Metabolic Disorders and Other Diseases

**DOI:** 10.3389/fcell.2022.811701

**Published:** 2022-02-14

**Authors:** Kihyoun Park, Myung-Shik Lee

**Affiliations:** Severance Biomedical Science Institute/Department of Internal Medicine, Yonsei University College of Medicine, Seoul, Korea and Soonchunhyang Institute of Medi-bio Science, Soonchunhyang University, Cheonan, Korea

**Keywords:** autophagy, modulator, metabolic diseases, lysosome, mitochondria, endoplasmic reticulum

## Abstract

Autophagy is pivotal in the maintenance of organelle function and intracellular nutrient balance. Besides the role of autophagy in the homeostasis and physiology of the individual tissues and whole organism *in vivo*, dysregulated autophagy has been incriminated in the pathogenesis of a variety of diseases including metabolic diseases, neurodegenerative diseases, cardiovascular diseases, inflammatory or immunological disorders, cancer and aging. Search for autophagy modulators has been widely conducted to amend dysregulation of autophagy or pharmacologically modulate autophagy in those diseases. Current data support the view that autophagy modulation could be a new modality for treatment of metabolic syndrome associated with lipid overload, human-type diabetes characterized by deposition of islet amyloid or other diseases including neurodegenerative diseases, infection and cardiovascular diseases. While clinically available bona fide autophagy modulators have not been developed yet, it is expected that on-going investigation will lead to the development of authentic autophagy modulators that can be safely administered to patients in the near future and will open a new horizon for treatment of incurable or difficult diseases.

## Introduction

Autophagy, derived from Greek words meaning “self-devouring,” is a cellular process of degradation of cell’s own material including organelles, proteins or cellular fluid in lysosome acting as the effector organelle of autophagic degradation (Mizushima and Komatsu, 2011; Agudo-Canalejo et al., 2021). Three major types of autophagy such as macroautophagy, microautophagy and chaperone-mediated autophagy have been described (Klionsky and Emr, 2000). Among them, macroautophagy (henceforth referred to as autophagy) is characterized by autophagosome which is encircled by double membrane assembled by subcellular membrane rearrangement sequestering organelles, target substrates and cytoplasm. After autophagosome fusion to lysosome, autophagolysosomes are formed in which sequestered material is decomposed or lysed by lysosomal enzymes (Klionsky and Emr, 2000). The main purpose of autophagy is quality control of organelles or proteins and protection of intracellular homeostasis or nutritional balance during energy deficiency. Thus, when nutrients are deficient, autophagy is initiated to mobilize intracellular nutrients and to avoid harmful effects caused by deficiency of vital nutrients or elements. When organelles are damaged, stressed or dysfunctional, autophagy receptors are recruited and act as bridges linking cargos to LC3 family proteins to initiate autophagic process. Detailed molecular and cellular mechanisms of the progression of autophagy after the initiation steps have been extensively investigated, and the Nobel Prize for Physiology or Medicine 2016 was bestowed on Yoshinori Ohsumi, as an accolade to his epoch-making discovery of the Atg conjugation system in autophagosome expansion.

Since autophagy is critical in the maintenance of cellular homeostasis and organelle function, autophagy affects diverse aspects of numerous physiological and pathological processes. Hence, dysregulated autophagy would lead to or be associated with a variety of diseases such as metabolic disorders including type 2 diabetes (T2D) or metabolic syndrome, neurodegenerative disorders including Alzheimer’s disease, Parkinson’s disease or Huntington’s disease, immune/inflammatory disorders, infectious diseases and cancer, etc. In T2D or metabolic syndrome, insulin and its downstream mTOR are well-known inhibitors of autophagy, while glucagon, a counter-regulatory hormone of insulin, is one of the first known inducers of autophagy (Deter and de Duve, 1967; Pfeifer, 1978; Sarbassov et al., 2005a). Furthermore, endoplasmic reticulum (ER) and mitochondria that play crucial roles in β-cell survival or function, insulin sensitivity or resistance and metabolic inflammation or inflammasome activation (Ozcan et al., 2004; Petersen et al., 2004; Laybutt et al., 2007; Misawa et al., 2013), rely on autophagy for proper function. Thus, autophagy is expected to affect diverse aspects of the pathogenesis of T2D or metabolic syndrome.

Because autophagy is involved in such various biological processes and diseases, searches for autophagy modulators have been conducted to develop novel compounds with therapeutic effects against those diseases or aging (Zhang et al., 2007; Eisenberg et al., 2009; Rubinsztein et al., 2012; Shoji-Kawata et al., 2013). In this review, the current development of autophagy modulators for clinical application will be summarized with a focus on therapeutic application to metabolic disorders. Since numerous papers are being published regarding autophagy enhancers for metabolic disorders and other diseases such as neurodegenerative diseases or cardiac disease, apologies are presented to the authors of the work which inadvertently was not covered in this review.

## Role of Autophagy in the Systemic Metabolism and Metabolic Disorders

**FIGURE 1:**
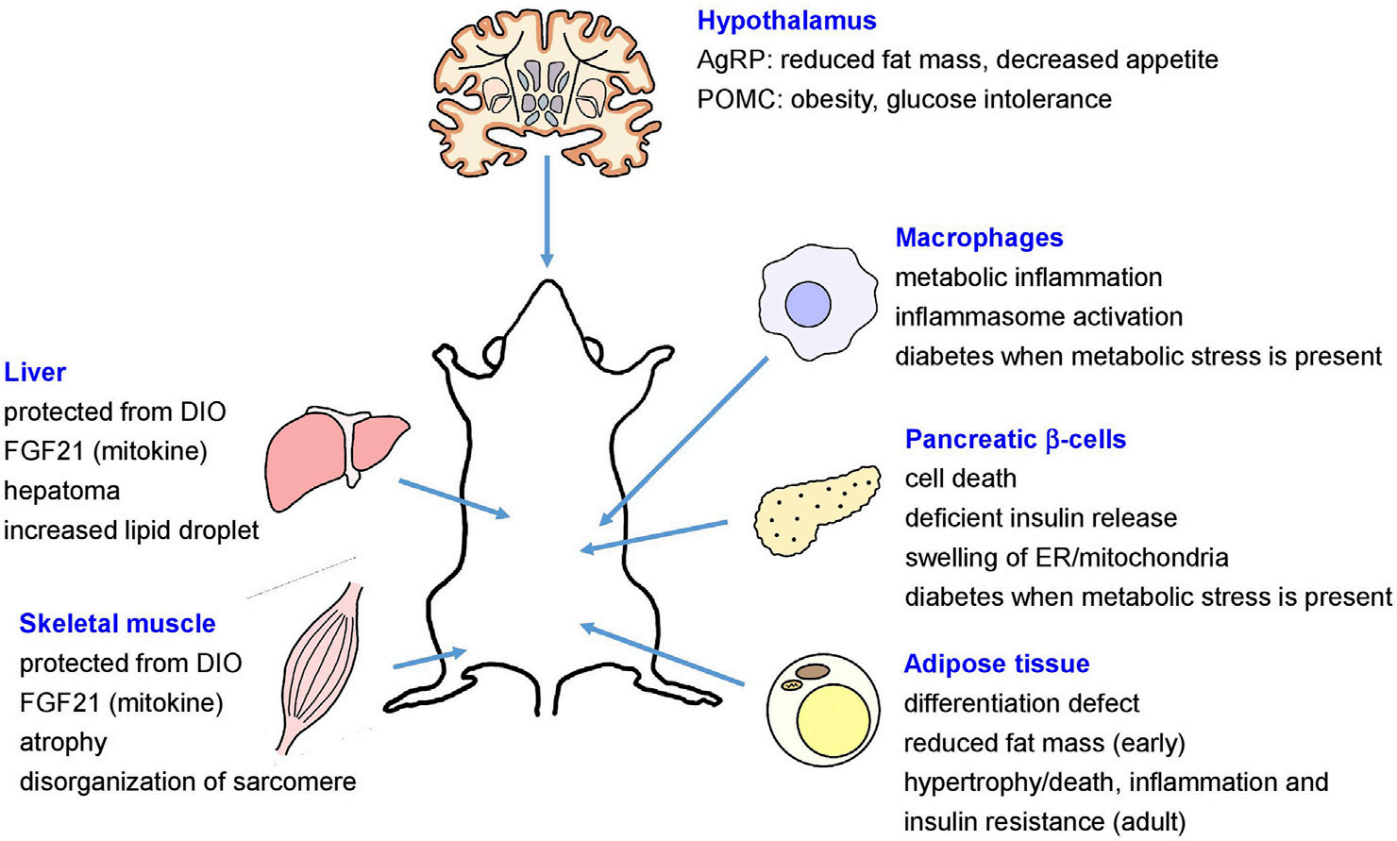
Metabolic effects of site-specific KO of autophagy genes (AgRP, agouti-related protein; DIO, diet-induced obesity; ER, endoplasmic reticulum; FGF21, fibroblast growth factor 21; POMC, proopiomelanocortin).

The role of autophagy in the maintenance of systemic or global metabolic homeostasis and in the pathogenesis of metabolic disorders has been controversial. *In vivo* role of autophagy in metabolic disorders has been mostly studied using genetic or site-specific autophagy gene-knockout (KO) mouse models that showed diverse metabolic features depending on the site of genetic alteration (Kim and Lee, 2014). For example, mice with KO of *Atg7*, an essential autophagy gene in pancreatic β-cells producing insulin, showed structural and functional defects of pancreatic β-cells, resulting in glucose intolerance and susceptibility to diabetes in the presence of metabolic stress (Ebato et al., 2008; Jung et al., 2008; Quan et al., 2012a) (Figure 1). On the contrary, KO of *Atg7* in insulin target tissues such as skeletal muscle cells or hepatocytes led to the induction of fibroblast growth factor 21 (FGF21) as a “mitokine” due to mitochondrial stress and to the resistance to diet-induced obesity and insulin resistance (Kim K. H. et al., 2013), contrary to the expectation that autophagy deficiency associated with mitochondrial dysfunction in insulin target tissues would lead to insulin resistance. In contrast, increased hepatic accumulation of lipid droplet has also been reported in mice with hepatocyte-specific *Atg5* KO or *Atg7* knockdown (Singh et al., 2009a; Yang et al., 2010) (Figure 1).

In mice with adipose tissue-specific KO of *Atg7*, defective differentiation of white adipocytes and reduced fat mass were observed (Singh et al., 2009b; Zhang et al., 2009). In contrast, adipose-specific KO of autophagy genes such as *Atg3, Atg16L1* or *Beclin 1* during adult period led to mitochondrial dysfunction, hypertrophic adipocytes due to impaired lipolysis, adipocyte death or inflammation in adipose tissue and peripheral insulin resistance or impaired glucose intolerance (Cai et al., 2018; Son et al., 2020) (Figure 1). Mice with deletion of *Atg7* in agouti-related protein (AgRP) neurons producing orexigenic hormones such as AgRP and neuropeptide Y (NPY) showed reduced fat mass and decreased hyperphagic response (Kaushik et al., 2011), while proopiomelanocortin (POMC) neuron-specific *Atg7*-KO mice showed obese phenotype and aggravated glucose intolerance after high-fat diet (HFD) feeding imposing metabolic stress (Coupe et al., 2012; Kaushik et al., 2012; Quan et al., 2012b) (Figure 1). Mice with macrophage-specific *Atg7* KO showed aggravated inflammasome activation in adipose tissue and metabolic deterioration after HFD feeding, which was associated with aggravated mitochondrial dysfunction (Kang et al., 2016; Lee et al., 2016) (Figure 1).

While these genetic models showed diverse metabolic phenotypes depending on the location and severity of autophagy deficiency, metabolic effects of systemic autophagy insufficiency of physiologically relevant degree rather than tissue-specific autophagy gene KO have been sparsely investigated. We have reported that global *Atg7*-haploinsufficient mice showed compromised adaptation to metabolic stress and accelerated progression from obesity to diabetes, which was associated with aggravated liver steatosis and augmented inflammasome activation in adipose tissue (Lim et al., 2014). In a similar vein, *Atg4b*-KO mice with reduced autophagic competence, exhibited aggravated metabolic profile after HFD (Fernández et al., 2017). Mice that are defective in stimulus-induced autophagy due to knock-in mutations in BCL2 phosphorylation sites (T69A, S70A and S84A) (*Bcl2 AAA* mice) also showed impaired exercise-mediated protection against glucose intolerance after HFD (He et al., 2012). On the other hand, overexpression of *Atg5* improved metabolic profile of aged mice (Pyo et al., 2013). These results indicate that adequate systemic autophagy is necessary for host adaptation to metabolic stress without which metabolic deterioration might ensue. These results also suggest the possibility that systemic enhancement of autophagic activity may confer beneficial metabolic effects against metabolic stress by enhancing cellular and global adaptive changes to the stress. In contrast, mice with genetic hyperactivation of autophagy through transgenic expression of constitutively active *Becn1* (*Becn1* ^F121A^) (Rocchi et al., 2017) displayed improved insulin sensitivity due to attenuated endoplasmic reticulum (ER) stress in peripheral tissues but aggravated glucose intolerance due to compromised β-cell function when fed HFD (Yamamoto et al., 2018). Such results have been ascribed to excessive degradation of insulin secretory granules by hyperactive β-cell autophagy, termed “vesicophagy,” and might suggest that intermittent rather than chronic activation of autophagy of an appropriate degree needs to be considered for treatment of T2D (Yamamoto et al., 2018).

While not an autophagy protein *per se*, effects of acyl-CoA binding protein (ACBP) involved in transport of acyl-CoA and lipid catabolism on autophagy and systemic metabolism has been studied. Upon autophagy induction, ACBP was released, and extracellular ABCP inhibited autophagy and promoted lipid anabolism or weight gain, which supports the view that systemic autophagy activation promotes lipid catabolism. On the other hand, intracellular ACBP stimulated autophagic flux, suggesting a feedback regulation of autophagy by ACBP, a lipid catabolic factor (Bravo-San Pedro et al., 2019).

## Autophagy in Human Diabetes or Murine Diabetes Mimicking Human Diabetes

Most of the studies investigating the role of autophagy in T2D or metabolic disorders have been employing genetic mouse models, and studies of the relationship between autophagy and metabolism in humans are sparse. Technological limitation in the assay of autophagic flux in human tissues or samples could be one of the reasons impeding studies of the role of autophagy in the metabolic regulation in humans. Previous studies of tissue samples from patients with T2D have examined morphological features and number of autophagosomes employing electron microscopy. In pancreatic islets of T2D patients, accumulation of enlarged autophagic vacuoles and autophagosomes was observed which was decreased after treatment with metformin, suggesting impeded removal of autophagic substances in islets of T2D patients (Masini et al., 2009). Protective effects of Imatinib-induced autophagy on human islet cell apoptosis by cytokine combinations have also been reported (Marselli et al., 2013). In human metabolic liver diseases, genetic association between non-alcoholic fatty liver disease (NAFLD) and variants of *IRGM*, an autophagy gene activated by infection (Grégoire et al., 2011) has been observed (Lin et al., 2016), suggesting the role of decreased autophagy-mediated lipid clearance or lipophagy in the development of human NAFLD.

Although most studies on the relationship between autophagy and metabolic diseases have been conducted using genetic mouse models as mentioned above, murine models of metabolic diseases cannot exactly recapitulate human metabolic diseases. Specifically, pathophysiological features of human diabetes and murine one are different in several aspects. One of the most striking differences is amyloid deposition in pancreatic islets of >90% of T2D patients but not in those of murine diabetes, which is due to different amino acid sequences of amylin or islet amyloid polypeptide (IAPP). Human IAPP (hIAPP) is amyloidogenic, while murine IAPP (mIAPP) is non-amyloidogenic because proline-rich sequence in residues 20–29 of mIAPP inhibits β-sheet formation, a prerequisite for amyloidogenesis (Westermark et al., 2011). It is unknown why hIAPP acquired amyloidogenic propensity during evolution. Because insoluble or amyloid proteins are preferentially cleared by autophagic pathway or lysosomal degradation due to their large size in contrast to soluble or non-amyloid proteins that can be cleared by both proteasomal and autophagic pathways (Rubinsztein, 2006), autophagy may be more important in human diabetes than in murine diabetes.

To study the role of autophagy in human-type diabetes characterized by islet amyloid accumulation, we have employed mice expressing *hIAPP* in pancreatic β-cells driven by rat insulin promoter (*hIAPP* ^+^ mice). While *hIAPP* ^+^ mice developed only mild hyperglycemia, *hIAPP* ^+^ mice crossed to β-cell-specific autophagy-KO mice (*hIAPP* ^+^ *Atg7* ^Δβ-cell^ mice) developed overt diabetes, accompanied by hIAPP oligomer and amyloid accumulation in pancreatic islets (Kim et al., 2014; Rivera et al., 2014). A study using *hIAPP* knock-in mice instead of *hIAPP* ^+^ mice also showed essentially similar results (Shigihara et al., 2014). These results demonstrate a critical role of β-cell autophagy in the clearance of hIAPP oligomer and in the prevention of islet amyloid deposition, which has implication in the understanding and treatment of human diabetes.

## Autophagy Enhancer in Metabolic Disorders

As the above results suggested potential therapeutic effects of autophagy modulation in T2D or metabolic syndrome and human-type diabetes, several autophagy enhancers have been developed or rediscovered from known drugs or chemicals against those diseases (Table 1).

**TABLE 1:** Autophagy enhancers that have beneficial effects on T2D or metabolic syndrome.

Drugs or chemicals	Mechanism and effects	Molecular targets	References	Clinical trial (phase)
Berberine	improved metabolic profile	AMPK or SIRT1	Ma, et al., 2018; Sun et al., 2018; Zheng et al., 2021	1, 2
Imatinib	Improved metabolic syndrome or diabetes	Kit or Abl;Beclin 1	Breccia et al., 2005; Han et al., 2009; Veneri et al., 2005; Lim et al., 2014	—
MSL-7	Enhanced clearance of lipid in the liver and amelioration of metabolic inflammation	TFEB	Lim et al. (2018)	—
	Enhanced clearance of hIAPP oligomer and improved β-cell function of *hIAPP* ^+^ mice on HFD		Kim et al. (2021b)	—
Rapamycin	Improved metabolic profile and β-cell function of Akita mice	mTOR	Bachar-Wikstrom et al. (2013)	—
	Increased longevity of mice		Harrison et al. (2009)	2
Resveratrol	Improved metabolic profile and extended healthspan of HFD-fed mice	SIRT1	Baur et al. (2006)	2
	Improved metabolic profile of patients with T2D		Bo et al. (2016)	2
Rg2	Improved glucose profile, insulin sensitivity and fatty liver change of HFD-fed mice	—	Fan et al. (2017)	—
Spermidine	Improved metabolic profile and liver steatosis in HFD-fed mice	EP300 eIF5A	Ma et al. (2021)	—
	Extended lifespan of *C. elegans*, *Drosophila* and aged mice		Eisenberg et al. (2009)	—
	Induction of hypusination of eIF5A and enhanced translation of TFEB		Zhang et al. (2019b)	—
Trehalose	Improved glucose profile and insulin sensitivity of obese mice	TFEB; AMPK	Lim et al. (2014)	—
	Decreased accumulation of hIAPP oligomer and amyloid in pancreatic islets		Kim et al. (2014)	—
	Improved fatty liver		Zhang et al. (2020c)	—
Triethylene-tetramine dihydrochloride (TETA)	improved metabolic profile of HFD-fed or *ob/ob* mice	SAT1	Castoldi et al. (2020)	—
Digoxin	Beneficial metabolic effects on metabolic syndrome after HFD; extension of *C. elegans* lifespan	TFEB	Wang et al. (2017)	1
Dioscin	Improved metabolic profile and liver steatosis of obese mice	—	Liu et al. (2015a)	—
Metformin	Protection of pancreatic β-cells against lipoapoptosis	AMPK	Jiang et al., 2014; Wu et al., 2013	3, 4
	Alleviation of aging-associated inflammation, fatty liver disease or diabetic kidney disease		Bharath et al., 2020; Ren et al., 2019; Song et al., 2015	3
GLP-1 agonist	Improved liver steatosis	AMPK or cGMP	Fang et al., 2020; Noyan-Ashraf et al., 2013; Sharma et al., 2011	4
	Improved diabetic nephropathy		Yamada et al. (2021)	4
SGLT2 inhibitor	Cardio- and reno-protective effects	AMPK	Packer, 2020a, Packer, 2020b	1, 4
PPAR-γ agonist	Protected β-cells against lipoapoptosis	AMPK	Wu et al. (2013)	—
	Ameliorated fatty liver disease		Hsiao et al. (2017)	4
PPAR-α agonist	Alleviated HFD-induced kidney injury in a diabetic kidney disease model	Autophagy genes	Sohn et al. (2017)	—
	Improved clinical course of clinical osteoarthritis in human patients		Nogueira-Recaldea et al. (2019)	—

Abbreviations: HFD, high-fat diet; TFEB, transcription factor EB.

### Berberine

**FIGURE 2:**
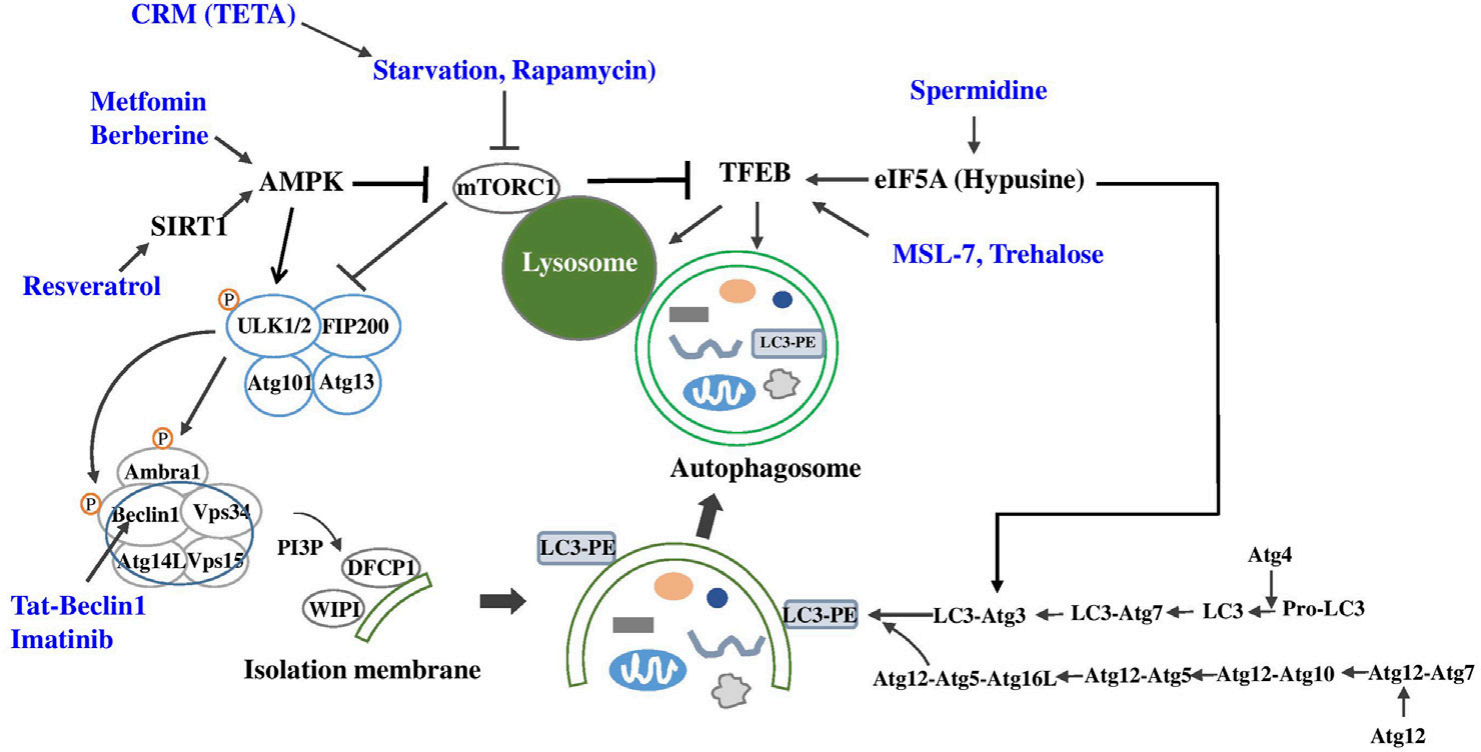
Autophagy enhancers for metabolic diseases (blue) and their target molecules (black) in autophagy machinery (CRM, calorie restriction mimic; TETA, Triethylenetetramine dihydrochloride).

Berberine, a traditional Chinese medicine, has been reported to improve metabolic profile of experimental animals or human patients with metabolic syndrome, diabetes or NAFLD which has been attributed to activation of AMPK and suppression of reactive oxygen species or inflammatory changes (Ma et al., 2018) (Figure 2). Recent papers showed the role of autophagy activation in the metabolic improvement by Berberine which was dependent on SIRT1 activation (Sun et al., 2018; Zheng et al., 2021). Autophagy-independent effects such as FGF21 induction has also been reported to contribute to the metabolic improvement by Berberine (Sun et al., 2018).

### Imatinib

Imatinib is a well-known anti-cancer drug that has a dramatic effect on chronic myelogenous leukemia (CML) and gastrointestinal stromal tumor by specifically inhibiting bcr-Abl and Kit kinases (Druker et al., 1996; Duensing et al., 2004). Imatinib has been reported to enhance autophagic activity of several cancer cells which may be related to the development of resistance against Imatinib. While conclusive mechanism of autophagy induction by Imatinib is still not clear, the mechanism of autophagy induction by Imatinib has been attributed to inhibition of Kit or Abl, increase of PARKIN-Beclin 1 interaction, induction of *Xiap, Atg5* or *Beclin 1*, and ER stress (Bellodi et al., 2009; Can et al., 2013; Etmer et al., 2007; Lin et al., 2015; Xie et al., 2017) (Figure 2). Imatinib can improve metabolic profile of experimental animals or patients with metabolic syndrome or diabetes (Breccia et al., 2005; Veneri et al., 2005; Han et al., 2009), which may be related to autophagy enhancement, while autophagy-independent mechanisms such as reduction of TNF-α release, ER stress or JNK activation might also contribute to the improved metabolic profile by Imatinib (Han et al., 2009). Furthermore, Imatinib could induce remission of diabetes of obese mice with insufficient autophagy, which was accompanied by autophagy induction *in vivo* and reduced insulin resistance (Lim et al., 2014). When Imatinib is considered as therapeutic agent inducing autophagic activity, potential adverse effects of Imatinib such as peripheral edema, cardiotoxicity and hematological side effects should be considered (Hartmann et al., 2009; Orphanos et al., 2009).

### MSL-7

We recently identified and developed autophagy enhancer small molecules in a screening of a chemical library. We observed that MSL can enhance nuclear translocation of TFEB, a principal regulator of autophagy gene expression and lysosome biogenesis (Settembre et al., 2011) in an mTORC1-independent manner (Lim et al., 2018) (Figure 2). We chemically modified MSL to enhance microsomal stability (MSL-7), and MSL-7 was able to exert beneficial metabolic effects on obese mouse models by enhancing the clearance of lipid in the liver tissue and ameliorating metabolic inflammation in adipose tissue (Lim et al., 2018). Attenuation of metabolic inflammation was due to amelioration of fatty acid-induced mitochondrial dysfunction and downregulation of fatty acid-induced inflammasome activation since mitochondrial events are important players in inflammasome activation (Misawa et al., 2013).

While these results showed improvement of metabolic profile of HFD-fed mice through enhanced clearance of lipid and reduced inflammasome activation, another potential metabolic effect of MSL-7 was studied because amyloidogenic hIAPP oligomer or islet amyloid (Westermark et al., 2011), is preferentially cleared by autophagy or lysosomal proteolysis rather than proteasomal degradation (Rubinsztein, 2006; Kim et al., 2014). Indeed, MSL-7 administration was able to improve glucose tolerance and β-cell function of HFD-fed *hIAPP* ^+^ mice expressing amyloidogenic *hIAPP* in β-cells, which was accompanied by reduction of hIAPP oligomer, islet amyloid or β-cell apoptosis and restoration of β-cell mass or insulinogenic index representing β-cell function (Kim et al., 2021b). These results suggest the possibility that autophagy enhancer may have more significant effects on human diabetes compared to its effects on murine diabetes because of additional effect of autophagy enhancer on β-cell viability and function.

### Rapamycin

Rapamycin is a classical autophagy enhancer acting through inhibition of mTOR (Klionsky and Emr, 2000) (Figure 2). mTOR can associate with other factors forming two mTOR complexes, mTORC1 and mTORC2. mTORC1 complex consists of mTOR, Raptor (regulator-associated protein of mTOR), PRAS40 (proline-rich Akt substrate, 40 kDa), Deptor and mLst8 (mammalian lethal with SEC13 protein 8), while mTORC2 complex comprises mTOR, Rictor, mSin1 (mammalian stress-activated protein kinase-interacting protein 1), Protor1/2, Deptor and mLst8 (Ardestani et al., 2018). It is generally held that mTORC1 is sensitive to rapamycin, whereas mTORC2 is not. However, prolonged rapamycin treatment has been shown to inhibit mTORC2 (Sarbessov et al., 2006).

Rapamycin has been administered to several models of diabetes. Rapamycin has been shown to improve metabolic profile and β-cell function of Akita mice with a mutation in proinsulin gene leading to proinsulin misfolding and severe ER stress, and such improved β-cell function was attributed to autophagy enhancement (Bachar-Wikstrom et al., 2013). However, prolonged administration of Rapamycin has deleterious effects on β-cell mass which is due to abrogation of mTORC1 activity positively modulating β-cell mass (Barlow et al., 2013). Prolonged Rapamycin administration can also impair insulin sensitivity, which could be due to inhibition mTORC2, since mTORC2 is PDK2 regulating Akt473 phosphorylation by insulin (Sarbassov et al., 2005b).

The effect of Rapamycin on longevity has been a controversial topic. Rapamycin was shown to prolong lifespan of mice (Harrison et al., 2009), while the role of autophagy in this model is unclear. In contrast to the effect of Rapamycin on longevity, Rapamycin had no clear effect on aging-related features such as kyphosis, gait disturbance, motor incoordination, impaired hippocampal neurogenesis, decreased grip strength, glomerulosclerosis or decreased CD4^+^ T cell number, suggesting that Rapamycin may prolong lifespan of mice independent of its effect on aging (Neff et al., 2013). In contrast, anti-aging effect or reversal of aging by Rapamycin such as reversal of reduced hematopoietic stem cell renewal, increased left cardiac ventricular mass and compromised myocardial performance or diastolic function in aged mice, have also been reported (Chen et al., 2009; Dai et al., 2014). In contrast to its effect on non-diabetic mice, Rapamycin shortened lifespan of diabetic *db/db* mice (Sataranatarajan et al., 2016).

### Resveratrol

Resveratrol, which is a natural polyphenol in red wine and well-known activator of SIRT1, has been reported to improve metabolic profile and extend healthspan of HFD-fed mice, which was associated with increased biogenesis of mitochondria and enhanced activities of PGC-1α or AMPK (Baur et al., 2006) (Figure 2). Resveratrol can promote autophagic activity through SIRT1-dependent deacetylation of autophagy proteins or TFEB (Morselli et al., 2011; Zheng et al., 2021). However, resveratrol administration for 6 months did not have beneficial metabolic effects in human patients with T2D (Bo et al., 2016). In contrast, small but significant effects of resveratrol on blood glucose, lipid profile or bone mineral density of patients with T2D have recently been reported (Bo et al., 2018; Hoseini et al., 2019).

### Rg2

Rg2, identified in a chemical screening using GFP-LC3 system, has been shown to improve glucose profile, insulin sensitivity and fatty liver change of mice fed HFD through autophagy activation (Fan et al., 2017). Rg2 also reduced body and fat weights of mice fed HFD; however, this effect on body weight was independent of autophagy.

### Spermidine

Spermidine, a polyamine synthesized *in vivo* from ornithine, has been reported to induce autophagy through epigenetic regulation of autophagy genes. Spermidine inhibits EP300 (E1A-binding protein p300), a lysine acetyltransferase and endogenous inhibitor of autophagy (Pietrocola et al., 2015). Spermidine has been reported to improve metabolic profile and liver steatosis of HFD-fed mice through autophagy activation, which was accompanied by reduced metabolic inflammation, enhanced thermogenesis and improved gut barrier function (Ma et al., 2021). However, autophagy-independent effects such as antioxidant effect or nitric oxide (NO) production might also play a role in the metabolic improvement by spermidine (Madeo et al., 2018).

In addition to the effect of spermidine on metabolic diseases, spermidine may affect aging. Given that spermidine level decreases in multiple organs by aging (Nishimura et al., 2006), decreased autophagic activity in aging (Hansen et al., 2018) may be causally related to spermidine insufficiency. Indeed, spermidine could extend longevity of *C. elegans* and *Drosophila* (Eisenberg et al., 2009). Furthermore, spermidine has also been reported to extend lifespan of aged mice by enhancing autophagy (Eisenberg et al., 2016). In a recent paper, spermidine has been shown to induce hypusination of eIF5A which is required for efficient translation of poly-proline proteins such as TFEB and ATG3, which led to the reversal of immune senescence of B lymphocytes (Zhang H. et al., 2019) and protection against premature brain aging or mitochondrial dysfunction (Liang et al., 2021) (Figure 2). The effect of spermidine on T cell autophagy has also been reported. Spermidine has been shown to enhance cytokine response of T cells from old vaccinees with suppressed autophagic activity by maintaining TFEB and autophagy (Alsaleh et al., 2020). It will be intriguing to investigate whether spermidine administration can reverse senescence or aging of other types of cells through similar mechanisms.

### Trehalose

Trehalose is a glucose dimer acting as a chemical chaperone, and has been reported to enhance autophagy. The mechanism of autophagy induction by trehalose has been attributed to inhibition of solute carrier 2A (SLC2A), a glucose transporter called GLUT, by trehalose, leading to activation of 5′ AMP-activated protein kinase (AMPK) and autophagy (DeBosch et al., 2016). However, since SLC2A inhibition was observed at high concentrations of trehalose above 10 mM, it is still not clear whether inhibition of glucose transporter can entirely explain autophagy activation by trehalose. Trehalose has also been reported to activate transcription of autophagy genes or transcription factor EB (TFEB), a master regulator of autophagy gene expression and lysosome biogenesis through low-grade lysosomal stress in an mTOR-independent FOXO1-dependent manner (Sarkar et al., 2007a; Dehay et al., 2010; Castillo et al., 2013; Jeong et al., 2021) (Figure 2).

Regarding therapeutic potential, trehalose has been shown to improve glucose profile and insulin sensitivity of obese mice with autophagy insufficiency, which was accompanied by amelioration of fatty liver (Lim et al., 2014). The effect of trehalose has also been tested in mice expressing amyloidogenic human-type *IAPP* fed HFD. In these mice, administration of trehalose improved glucose profile and augmented insulinogenic index representing β-cell function. Accumulation of hIAPP oligomer and amyloid in pancreatic islets was also attenuated by trehalose (Kim et al., 2014). These results suggest possible therapeutic potential of autophagy enhancers in human-type diabetes.

Trehalose is degraded by intestinal trehalase, which may affect bioavailability of ingested trehalose. Lentztrehalose, a novel analog of trehalose with reduced susceptibility to trehalase, has been produced (Wada et al., 2015). Lactotrehalose, another trehalase-resistant, has been shown to ameliorate fatty liver after high-sucrose diet feeding (Zhang et al., 2020b). Contrary to these reports showing autophagy-enhancing effect of trehalose, a previous paper has questioned autophagy enhancing activity of trehalose (Kaizuka et al., 2016).

### Triethylenetetramine Dihydrochloride

TETA, a Cu^2+^-chelating agent that has been used for treatment of Wilson’s disease, has been shown to improve metabolic profile of HFD-fed or *ob/ob* mice. Such metabolic effects were related not to chelation of Cu^2+^ but to stabilization and increased activity of spermidine N^1^-acyltransferase (SAT1) that is also necessary for beneficial effects of spermidine on host metabolism (Castoldi et al., 2020) (Figure 2 **)**.

### Other Autophagy Enhancers in Metabolic Diseases

Digoxin; ikarugamycin, a natural marine-derived product; and alexidin dihydrochloride, a synthetic compound, have been identified in a screening for TFEB agonists and have been reported to have beneficial metabolic effects on metabolic syndrome after HFD feeding and to extend lifespan of *C. elegans* (Wang et al., 2017). Dioscin, a natural steroid saponin, has been reported to improve metabolic profile and liver steatosis of obese mice through autophagy upregulation (Liu M. et al., 2015).

### Known Drugs Against T2D or Other Metabolic Diseases

Some of the effects of drugs that are already being used in the clinic against T2D or other metabolic diseases have been ascribed to autophagy enhancement. Such drugs may work through both the originally proposed mechanisms and autophagy modulation, while the proportion of autophagy modulation in the pharmacological effects of the drug could be different from one drug to another.

#### Metformin

Metformin is a widely-used anti-diabetic medicine that has been recommended as the first-line drug against diabetes. While metformin has AMPK-independent mechanisms for improvement of metabolic profile (Kalender et al., 2010), most investigators concur with the opinion that metformin activates AMPK (Zhou et al., 2003). Then, metformin would be able to enhance autophagic activity since AMPK activation can upregulate autophagic activity through direct phosphorylation of ULK1 and Beclin 1, key molecules in the initiation of autophagy, or inhibition of mTORC1 (Egan et al., 2011; Howell et al., 2017; Kim J. et al., 2013; Kim et al., 2011) (Figure 2). Autophagy induction by AMPK activation is in line with the concept that autophagy is an adaptive process in response to nutrient deficiency and that AMPK is a sensor of intracellular energy balance. Hence, well-established AMPK activation by metformin suggests the possibility that improvement of metabolic profile by metformin might be related to autophagy induction through AMPK activation. Consistent with this concept, protection of pancreatic β-cells against lipoapoptosis by metformin was attributed to autophagy activation (Wu et al., 2013; Jiang et al., 2014). Metformin has also been shown to enhance disposal of accumulated autophagic vacuoles in β-cells (Masini et al., 2009), and to alleviate aging-associated inflammation, fatty liver disease or diabetic kidney disease through autophagy induction (Song et al., 2015; Ren et al., 2019; Bharath et al., 2020). Thus, it is likely that anti-diabetic effect of metformin could be partly due to autophagy activation, in addition to autophagy-independent effects such as inhibition of mitochondrial complex I activity or alteration of gut microbiota (Owen et al., 2000; Shin et al., 2014).

#### GLP-1 Agonist

GLP-1 receptor agonists such as liraglutide have been reported to enhance autophagy level of insulinoma cells (Chen et al., 2013). While GLP-1 receptor agonists may upregulate autophagy in several tissues including the liver and thereby improve liver steatosis (Sharma et al., 2011; Noyan-Ashraf et al., 2013; Fang et al., 2020), the mechanism of autophagy induction by of GLP-1 receptor or other G-protein-coupled receptors (GPCR) is unclear (Wauson et al., 2014). In this regard, role of increased AMPK or cGMP and reduced mTOR or GSK3β signaling have been suggested (Candeias et al., 2018; Yang et al., 2018). Increases of autophagy by GLP-1 receptor agonists might also be related to the amelioration of ER stress (Yusta et al., 2006).

Additionally, GLP-1 agonists have been reported to have therapeutic effects on diabetic kidney disease (Yamada et al., 2021) through promotion of autophagy.

#### SGLT2 Inhibitor

Sodium-glucose transporter 2 (SGLT2) inhibitor is a novel class of anti-diabetic drug acting by increasing urinary loss of glucose and inducing a starvation-like state. SGLT2 inhibitors have been shown to enhance autophagic activity through AMPK activation (Hawley et al., 2016). SGLT2 inhibitors have also been reported to induce autophagy in diabetic heart and kidney tissues, which might be related to cardio- or reno-protective effects of SGLT2 inhibitors (Packer, 2020a; Packer, 2020b).

#### PPAR-γ Agonist

Peroxisome proliferator-activated receptor γ (PPAR-γ) is a transcriptional factor regulating expression of genes involved in adipogenesis and lipid uptake (Spiegelman, 1998). Rosiglitazone, a representative PPAR-γ agonist, has been reported to protect β-cells against lipoapoptosis, which was ascribed to the activation of autophagy through AMPK (Wu et al., 2013). On the other hand, inhibition of autophagy by rosiglitazone has also been reported (Ji et al., 2018; Li et al., 2018).

Pioglitazone, another PPAR-γ agonist, has also been reported to ameliorate fatty liver disease through autophagy activation (Hsiao et al., 2017).

#### PPAR-α Agonist

PPAR-α is a transcriptional factor which plays a crucial role in the expression of genes involved in fatty acid oxidation, lipid transport and ketosis (Torra et al., 2000). PPAR-α is induced by fasting (Kersten et al., 1999), suggesting its potential relationship with autophagy induction by fasting. Indeed, PPAR-α has been shown to bind promoters of several autophagy genes. Furthermore, GW7647, a PPAR-α agonist, has been reported to reverse feeding-induced autophagy suppression (Lee et al., 2014). Fenofibrate, a classical PPAR-α agonist, has been reported to alleviate HFD-induced kidney injury in a diabetic kidney disease model probably through autophagy activation (Sohn et al., 2017). While not directly related to diabetes or metabolic diseases, fenofibrate has also been reported to suppress cartilage degeneration *in vitro* through autophagy activation, and use of fenofibrate has been associated with improved clinical course of osteoarthritis in human patients (Nogueira-Recalde et al., 2019).

On the contrary, FXR agonist was reported to compete with PPAR-α for binding to promoters of autophagy genes and to suppress autophagy (Lee et al., 2014).

## Autophagy Modulators in Other Diseases

Autophagy modulators have been developed for diseases other than metabolic disorders (Table 2).

**TABLE 2:** Autophagy enhancers that have beneficial effects on diseases other than metabolic disorders.

Drugs or chemicals	Mechanism and effects	Molecular targets	Target diseases	References	Clinical trial (phase)
AUTEN-67	Retarded disease progression	MTMR14	Huntington’s disease or Alzheimer’s disease	Billes et al., 2016; Papp et al., 2016	—
EN6	Accelerated clearing of TDP-43 aggregates	ATP6V1A	ALS	Chung et al. (2019)	—
ERRα Inverse Agonist	Enhancement of autophagosome fusion to lysosome	Autolyso-some	Parkinson’s disease	Suresh et al. (2018)	—
HTT Linker	Ameliorated disease phenotype	LC3	Huntington’s disease	Li et al. (2019)	—
ML246	Improved memory	Beclin 2	Alzheimer’s disease	Rocchi et al., 2017; Kuramoto et al., 2016	—
ML-SA1	Protection of dopaminergic neurons	TFEB	ALS and Parkinson’s disease	Tedeschi et al., 2019; Tsunemi et al., 2019	—
	Reduced apoptosis of photoreceptor cells		Retinal detachment	Yan et al. (2021)	—
Nilotinib	Degradation of α-synuclein and reversion of dopaminergic neuron loss	Beclin 1, Atg12	Parkinson disease	Hebron et al. (2013)	1,2
SMER	Increased clearance of mutant huntingtin or α-synuclein	—	Huntington’s disease	Sarkar et al. (2007b)	—
	Attenuated neurodegeneration		Alzheimer’s disease	Tian et al. (2011)	—
	Increased erythropoiesis		Diamond-Blackfan syndrome	Doulatov et al. (2017)	—
Spermidine	Improved cognition	PARKIN	Aging	Lazarou et al., 2015; Schroeder et al., 2021	—
	Improved cardiac function		Heart failure	Eisenberg et al. (2016)	—
Tat-beclin 1	Improved cognitive function	GAPR-1	Alzheimer’s disease	Shoji-Kawata et al. (2013)	—
	Beneficial effects on cardiac function after TAC		Heart failure	Shirakabe et al. (2016)	—
	Reduced microbial replication and improved clinical outcome of infection		*Listeria* monocytogenes; Sindvis virus, chikungunya virus, West Nile virus, HIV	Shoji-Kawata et al. (2013)	—
Trehalose	Reduced Aβ deposition and improved cognitive deficit and learning disability	TFEB	Alzheimer’s disease	Du et al. (2013)	1
	Reduced atherosclerotic plaque burden in *ApoE*-KO mice fed western diet		Atherosclerosis	Sergin et al. (2017)	2
	Attenuated inflammation of the brain		Mucopolysacchari-dosis IIIb	Lotfi et al. (2018)	—
UMI-77	Ameliorated neurological deficit	MCL-1	Alzheimer’s disease	Cen et al. (2020)	—
Felodipine	Neuroprotection	—	Huntington’s disease	Siddiqi et al. (2019)	—
Rilmenidine	Amelioration of neurological signs	—	Huntington’s disease	Rose et al. (2010)	—
Lithium	Clearance of α-synuclein aggregate and mutant Huntingtin	Inositol monophos-phatase	Parkinson’s disease; Huntington’s disease	Sarkar et al. (2005)	1, 2
GLP-1 agonists	Improved motor function	mTOR	Parkinson disease	Zhang et al. (2020a)	—
	Attenuated fibrosis after aortic banding		Heart Diseases	Zheng et al. (2019)	—
PPAR-α agonist	Improved cognition	Autophagy genes	Alzheimer’s disease	Luo et al. (2020)	1
Sildenafil	Improved hear failure	PGK1	Heart Diseases	Ranek et al. (2019)	4
Metformin	Ameliorated ultrastructural abnormalities	AMPK	Diabetic Heart Diseases	Xie et al. (2011)	
	Autophagy induction		Diabetic kidney disease	Kaushal et al. (2020)	4
Fenofibrate	Prevention of fibrosis in an animal model of T1D	Autophagy genes	Heart Diseases	Zhang et al. (2016)	—
Artemisinin	Alleviation of atherosclerosis	AMPK	Atherosclerosis	Cao et al. (2019)	—
Fucoidan	Alleviation of atherosclerosis	TFEB	Atherosclerosis	Cheng et al. (2020)	—
Rapamycin	Ameliorated damage after coronary ligation	mTOR	Ischemic heart disease	Sciarretta et al. (2012)	—
Rapamycin	Suppressed photoreceptor degeneration or inflammation	mTOR	Retinitis	Okamoto et al. (2016)	—
Temsirolimus	Protected against sepsis-induced acute renal failure	mTOR	Kidney Diseases	Howell et al. (2013)	2
Urolithin A	Ameliorated I/R-induced injury	TFEB	Kidney Diseases	Wang et al. (2019b)	
Atrasentan	Amelioration of diabetic kidney disease and enhancement of Foxo1 expression	miR-21	Kidney Diseases	Wang et al. (2019a)	2
Klotho	Ameliorated FK506-induced injury	TFEB	Kidney Diseases	Lim et al. (2019)	—
A77 1726	Restriction of bacterial growth	AMPK	Infection (*Salmonella*)	Zhuang et al. (2020)	—
Flubendazole	Promoted clearance of intracellular bacteria	mTOR	Infection (HIV)	Chauhan et al. (2015)	—
BC18630	Reduced viral load and lung injury	TFEB	Infection (SARS-CoV-2)	Liu et al. (2021)	—
2-hydroxypropy l-β-cyclodextrin	Accelerated clearance of proteolipid aggregates	TFEB	Neuronal ceroid lipofuscinosis	Song et al. (2014)	—
Rosiglitazone	Attenuated post-operative fibrosis	Beclin 1	Glaucoma	Zhang et al. (2019a)	—
Artesunate (ART)	Ameliorated retinopathy	AMPK/SIRT1	Diabetic retinopathy	Li et al. (2021)	—
Apigenin	Improved clinical index and reduce intraocular oxidative damage	Nrf2	Age-related macular degeneration	Zhang et al. (2020b)	—
Latrepirdine	Improved cognition	mTOR	Alzheimer’s disease	Steele and Gandy, (2013)	—
Calpastatin	Improved motor function and decrease tremor	Calpain	Huntington’s disease	Menzies et al. (2015)	—
6-Bio	Improved motor coordination and locomotion	GSK3b	Parkinson’s disease	Suresh et al. (2017)	—
Vitamin D	Inhibited viral replication	Beclin 1	HIV infection	Campbell and Spector, (2011)	2, 3
Acacetin	Reduced bacterial burden *in vivo*	TFEB	*Salmonella*	Ammanathan et al. (2020)	—

Abbreviations: ALS, amyotrophic lateral sclerosis; T1D, Type 1 diabetes; TAC, Transverse aortic constriction.

### Neurodegenerative Diseases

In the pathogenesis of many neurodegenerative diseases, accumulation of amyloidogenic, aggregate or misfolded proteins plays a critical role. Since such proteins are preferentially cleared by autophagy or lysosomal degradation pathway rather than proteasomal degradation pathway, potential relevance of autophagy modulators as future therapeutic agents against neurodegenerative diseases is immense (Rubinsztein, 2006). Defective autophagy or lysosomal dysfunction has been demonstrated to play a crucial pathogenic role in diverse neurodegenerative diseases such as Parkinson’s disease or Alzheimer’s disease (Nixon, 2007; Narendra et al., 2008; Bordi et al., 2016). In addition, aging, one of the most important risk factors for neurodegenerative diseases such as Alzheimer’s disease (Thelen and Brown-Borg, 2020), diminishes autophagic activity in multiple tissues in a variety of non-mammalian and mammalian species (Sun et al., 2015; Hansen et al., 2018). For instance, the role of autophagy in aging or longevity is clearly seen in lower organisms such as *C. elegans* since knockdown of *Beclin 1* reduced lifespan of *C. elegans* (Melendez et al., 2003). In higher or vertebrate organisms, the role of autophagy in aging is less clear. However, a couple of papers suggested an important role of autophagy in aging or longevity of mammals. Overexpression of an autophagy gene has also been reported to prolong lifespan of mice and to improve metabolic profile of aged mice (Pyo et al., 2013). Furthermore, autophagic or mitophagic activity and lysosomal function decline with aging (Hütter et al., 2007; Salminen and Kaarniranta, 2009; Sun et al., 2015; Fernando et al., 2020), while the details of the changes could be different depending on the tissue and species. These results suggest that autophagy could be intimately related to aging of mammals as well.

Given the role of autophagy deficiency or lysosomal dysfunction in the pathogenesis of neurodegenerative disorders and the close relationship between autophagy or lysosomal function and aging, autophagy enhancers could have beneficial effects against diverse neurodegenerative disorders as discussed below. Further detailed discussion regarding the role of autophagy specifically in neurodegenerative diseases can be found in recent review articles (Rahman and Rhim, 2017; Park et al., 2020). Several autophagy enhancers against neurodegenerative diseases not covered in the text can be found in Table 2.

#### AUTEN-67

AUTEN-67 (Autophagy Enhancer-67), an MTMR14 inhibitor, has been reported to impede progression of the diseases in a *Drosophila* model of Huntington’s disease and in a mouse model of Alzheimer’s disease (Billes et al., 2016; Papp et al., 2016).

#### EN6

A small molecule autophagy activator (EN6) that covalently targets cysteine 277 of ATP6V1A subunit of lysosomal v-ATPase was identified in a screening using cysteine- and lysine-reactive ligands. EN6 decoupled v-ATPase from the Ragulator-Rag complex and induced mTORC1 inhibition, leading to accelerated clearing of TDP-43 aggregates through autophagy enhancement (Chung et al., 2019).

#### ERRα Inverse Agonist

It was reported that overexpression of ERRα inhibits autophagosome fusion to lysosome (Suresh et al., 2018). XCT 790, an inverse agonist of ERRα, was shown to exert neuroprotective effects in a preclinical model of Parkinson’s disease through autophagy upregulation (Suresh et al., 2018).

#### HTT Linker

Recently, compounds linking target substrates to LC3 have been developed. An HTT-LC3 linker has been reported to ameliorate Huntington’s disease phenotype by tethering mutant Huntingtin to LC3 (Li et al., 2019). This compound belongs to the class of autophagosome tethering compounds (ATTECs).

#### ML246

ML246, a brain-permeable autophagy enhancer, has been shown to enhance clearance of Aβ, protect neuronal cells from Aβ-induced cell death, and improve memory of Alzheimer’s disease mice, which was associated with a trend toward reduced brain amyloid accumulation (Rocchi et al., 2017). ML246 has also been shown to prevent cannabinoid tolerance by inhibiting Beclin 2 (BECN2) binding to GRASP1, a receptor for cannabinoid receptor 1 (CNR1) degradation (Kuramoto et al., 2016), which might be employed to enhance medical effects of cannabinoid such as analgesia.

#### ML-SA1

Transient receptor potential cation channel, mucolipin subfamily 1 (TRPML1) channel is a lysosomal Ca^2+^ exit channel that is important in activation of TFEB, a master regulator of autophagy gene expression and lysosomal biogenesis (Wang et al., 2015). ML-SA1, an agonist of TRPML channel (Shen et al., 2012), has been reported to protect dopaminergic neurons in cell models of amyotrophic lateral sclerosis (ALS) and Parkinson’s disease by boosting autophagy or enhancing lysosomal Ca^2+^ exocytosis (Tedeschi et al., 2019; Tsunemi et al., 2019).

#### Nilotinib

As an Abl inhibitor related to aforementioned Imatinib, Nilotinib has been shown to induce degradation of α-synuclein and reverse loss of dopaminergic neurons through autophagy activation which was associated with increased levels of Beclin 1 and Atg12 (Hebron et al., 2013). Radotinib, a related compound, has also been reported to be neuroprotective in a preclinical Parkinson disease model (Lee et al., 2018). However, recent phase 2 clinical trials using Nilotinib showed mostly negative results (Simuni et al., 2021). A recent paper also reported no effect of Nilotinib on R6/2, an animal model of Huntington’s disease (Kumar et al., 2021).

#### SMER

SMERs were initially selected by small-molecule screening in yeast and, their activity on mammalian autophagy was confirmed employing a cell system overexpressing A53T α-synuclein, an autophagy substrate. SMER10 (aminopyrimidone), SMER18 (vinylogous amide), SMER28 (bromo-substituted quinazoline) and their analogs increased autophagic clearance of mutant huntingtin or α-synuclein *in vitro* in an-mTOR-independent manner, and also attenuated neurodegeneration in *D. melanogaster* model of Huntington’s disease (Sarkar et al., 2007b). Later studies showed that SMER28 can accelerate *in vitro* clearance of Aβ peptide or carboxy terminal fragment of amyloid precursor protein (APP-CTF) (Tian et al., 2011). Besides neurodegenerative disorders, SMER28 has been reported to increase erythropoiesis in cell or animal models of Diamond-Blackfan syndrome, a disorder of abnormal erythroid progenitor differentiation, through *Atg5* (Doulatov et al., 2017).

#### Spermidine

Aforementioned spermidine was recently reported to improve cognitive dysfunction in aged mice, which was associated with increased mitochondrial respiration and dependent on *Atg7* and *PINK1*, a kinase inducing mitophagy through recruitment of PARKIN onto dysfunctional mitochondria (Lazarou et al., 2015; Schroeder et al., 2021).

#### Tat-Beclin 1

HIV Nef-interacting domain of Beclin 1 attached to Tat transduction domain (Tat-beclin 1) could enhance autophagic flux by binding to GAPR-1, an autophagy inhibitor, and liberating Beclin 1 from Golgi complex (Shoji-Kawata et al., 2013) (Figure 1). Tat-beclin 1 has been reported to accelerate clearance of htt103Q aggregate or Aβ oligomer and to improve cognitive function of Alzheimer’s disease model mice (Shoji-Kawata et al., 2013). In contrast, a high dose of Tat-beclin 1 has been reported to induce autosis, a special form of cell death characterized by high autophagic activity, inhibition by Na^+^/K^+^ ATPase inhibitors and unique morphological features including focal rupture of plasma membrane, electron-dense swollen mitochondria and ballooning of perinuclear space (Liu et al., 2013). Furthermore, Tat-Beclin 1 peptide has been shown to aggravate hypoxic injury of the rat brain, which might be related to the inhibition of autophagosome-lysosome fusion due to elevated expression of Rubicon (Nah et al., 2020).

#### Trehalose

Trehalose, a disaccharide with autophagy enhancing activity as discussed above, was also shown to exert effects on neurodegeneration and aging. Trehalose could prolong total and reproductive life span, retard decrease in pharyngeal pumping due to aging, enhance thermotolerance and suppress polyQ aggregation in *C. elegans*, while the role of autophagy in trehalose effects was not directly demonstrated in this paper (Honda et al., 2010). Trehalose also reduced Aβ deposition and improved cognitive deficit and learning disability in a mouse model of Alzheimer’s disease (Du et al., 2013).

#### UMI-77

In a screening using an FDA-approved drug library, UMI-77, a BH3-mimetic for MCL-1, was identified as an inducer of mitophagy without effects on apoptosis. MCL-1 was suggested to be a mitophagy receptor, and UMI-77 ameliorated neurological deficit in a mouse model of Alzheimer’s disease (Cen et al., 2020).

#### Other Previously Known Chemicals or Drugs

In several screening for autophagy modulators, some known chemicals or drugs have been identified and shown to enhance clearance of aggregate or amyloid proteins associated with neurodegeneration. For instance, GFP-LC3-based screening followed by assay of FYVE-RFP^+^ vesicle identified eight known chemicals inducing autophagic degradation of long-lived proteins in an mTOR-independent manner (Fluspirilene, trifluoperazine, pimozide, nicardipine, penitrem A, niguldipine, loperamide and amiodarone). Those chemicals also reduced accumulation of polyQ (Zhang et al., 2007).

Another autophagy enhancer screening using EGFP-LC3 vesicles identified verapamil, valproate and clonidine as autophagy enhancers acting through decreased (Ca^2+^)_i_ or intracellular inositol 1,4,5-trisphosphate (IP_3_) content and calpain inhibition (Williams et al., 2008). Such chemicals have been reported to delay Huntington’s disease-like phenotypes in *Drosophila* or zebrafish models. A similar screening of autophagy enhancer using GFP-RFP-LC3 system and a chemical library identified flubendazole, an anti-helminthic agent, and bromhexine as autophagy enhancers. Flubendazole led to disrupted dynamic microtubule, followed by displacement and inhibition of mTOR. Flubendazole and bromhexine were also reported to reduce accumulation of total and hyperphosphorylated Tau (Chauhan et al., 2015). Felodipine, an anti-hypertensive drug acting on L-type Ca^2+^ channel similar to verapamil, has also been reported to have autophagy-enhancing and neuroprotective effects in animal models of Huntington’s disease (Siddiqi et al., 2019). Rilmenidine, another anti-hypertensive drug, has been reported to enhance autophagy and to ameliorate neurological signs in an animal model of Huntington’s disease (Rose et al., 2010).

Lithium, a well-known drug for bipolar disease, has been reported to induce autophagy through inhibition of inositol monophosphatase and depletion of free inositol or IP_3_. Lithium has been shown to induce clearance of α-synuclein aggregate or mutant Huntingtin (Sarkar et al., 2005). A recent paper showed the effect of lithium in the clearance of Tau aggregate (Uddin et al., 2021).

GLP-1 agonists, aforementioned anti-diabetic drugs with potential autophagy enhancing activity, have also been reported to confer therapeutic effects on Parkinson disease animal models (Zhang L. et al., 2020).

PPAR-α agonist such as gemfibrozil or Wy14643 has been reported to ameliorate Alzheimer’s disease-related phenotype in a murine model through autophagy induction (Luo et al., 2020).

#### Heart Diseases

Autophagy is crucial in the development of the heart and in the maintenance of cardiac function (Gatica et al., 2021). Dysregulated autophagy has been reported to be involved in the pathogenesis of several cardiovascular diseases.

As discussed above, spermidine induced autophagy through epigenetic regulation of autophagy genes (Pietrocola et al., 2015), and could extend lifespan of experimental animals (Eisenberg et al., 2016). Spermidine has also been reported to improve cardiac function in age- or hypertension-associated heart failure models (Eisenberg et al., 2016).

Sildenafil, an inhibitor of phosphodiesterase type-5, can activate protein kinase G1 (PGK1) that has been shown to phosphorylate TSC2 and induce autophagy through mTORC1 inhibition (Ranek et al., 2019). Sildenafil has been reported to reverse autophagy inhibition and heart failure after cardiac pressure overload in a phosphorylation-silencing *Tsc2* mutant-knockin mouse model (Ranek et al., 2019).

Everolimus, a relatively selective inhibitor of mTORC1, also had similar effects on failing heart in the same mice.

Metformin, a well-known anti-diabetic drug, has been reported to enhance cardiac autophagic activity and ameliorates cardiac ultrastructural abnormalities associated with diabetes through AMPK activation in an animal model of diabetic cardiomyopathy (Xie et al., 2011).

GLP-1 agonists have also been reported to have therapeutic effects on myocardial fibrosis after aortic banding through autophagy enhancement (Zheng et al., 2019).

Fenofibrate, a classical PPAR-α agonist, has been reported to prevent cardiac fibrosis in an animal model of type 1 diabetes (T1D), accompanied by enhanced autophagic activity *in vivo* (Zhang et al., 2016).

Tat-Beclin 1, aforementioned autophagy enhancer liberating beclin 1 from Golgi complex, was shown to confer beneficial effects on heart failure after transverse aortic constriction (TAC) (Shirakabe et al., 2016), which is consistent with a previous report that cardiac autophagy plays a protective role in ischemic cardiac disease of mice with diet-induced obesity (Sciarretta et al., 2012). On the other hand, Tat-Beclin 1 peptide has been shown to aggravate cardiac ischemia-reperfusion (I/R)-induced cardiac injury and autosis, which was accompanied by increased expression of Rubicon inhibiting multiple steps of autophagy including autophagosome-lysosome fusion (Nah et al., 2020).

Another example showing deleterious effect of autophagy on cardiac tissue has been published. A paper reported that dimethyl α-ketoglutarate inhibited mal-adaptive cardiac autophagy and suppressed TAC-induced heart failure in a pressure-overload-induced cardiomyopathy model after conversion to cytosolic acetyl CoA acting on EP300, an acetyltransferase (Mariño et al., 2014).

In addition to heart failure, atherosclerosis is another heart disease that is closely related to autophagy, particularly that of macrophages (Liao et al., 2012). Trehalose, an aforementioned autophagy enhancer, could reduce atherosclerotic plaque burden in *ApoE*-KO mice fed western diet by enhancing autophagy (Sergin et al., 2017).

Spermidine, an autophagy enhancer discussed above, has also been reported to attenuate atherosclerosis and to inhibit necrotic core formation through autophagy activation (Michiels et al., 2016).

Artemisinin is an endoperoxide sesquiterpene lactone with multiple beneficial effects such as anti-malarial, anti-inflammation and anti-oxidant effects. Artemisinin has also been reported to attenuate atherosclerosis in HFD-fed *ApoE*-KO mice by enhancing autophagic activity of macrophages (Cao et al., 2019).

Fucoidan, a marine sulfated polysaccharide derived from brown seaweeds with anti-inflammatory activity, has also been reported to alleviate atherosclerosis in HFD-fed *ApoE*-KO mice by enhancing autophagic activity (Cheng et al., 2020).

In ischemic heart disease, Rapamycin, a classical mTOR inhibitor and autophagy enhancer, could ameliorate cardiac damage after coronary ligation in mice fed HFD by enhancing autophagy and protecting against cardiomyocyte death (Sciarretta et al., 2012).

Further detailed discussion regarding the role of autophagy specifically in cardiovascular diseases can be found in recent review articles (Lampert and Gustafsson, 2018; Gatica et al., 2021)

### Kidney Diseases

Kidney is a critical target organ of diabetic complication. Previous papers showed the role of altered autophagy in the development of several kidney diseases including diabetic nephropathy and kidney fibrosis (Liu W. J. et al., 2015; Nam et al., 2019; Zhao et al., 2019). Several autophagy enhancers have been administered to such diverse kidney disease models.

Temsirolimus, an autophagy enhancer belonging to mTORC1 inhibitor family, has been administered to septic kidney disease model with beneficial clinical effects accompanied by autophagy enhancement (Howell et al., 2013). A variety of authentic or potential AMPK activators such as metformin, ω-3 polyunsaturated fatty acids, quercetin, neferine, astragaloside IV, mangiferin, cinacalcet, berberine, progranulin have also been administered to diabetic kidney disease, cisplatin-induced nephropathy or I/R kidney injury, leading to clinical improvement and autophagy enhancement (Kaushal et al., 2020).

Aforementioned autophagy enhancers such as fenofibrate, pioglitazone and SGLT2 inhibitors have also been employed to treat diabetic kidney disease or I/R kidney injury with clinical improvement.

Urolithin A, the main metabolite in pomegranate juice, has been reported to ameliorate I/R-induced kidney injury through TFEB activation (Wang Y. et al., 2019).

Atrasentan, an antagonist of endothelin 1 receptor subtype A (ET_A_), was able to ameliorate diabetic kidney disease, by downregulating miR-21 expression, enhancing Foxo1 expression and upregulating autophagy (Wang J. et al., 2019).

Klotho, an obligate co-receptor for fibroblast growth factor 23 (FGF23), has also been reported to ameliorate FK506-induced renal injury with impaired lysosomal function by inducing TFEB activation through inhibition of GSK3β phosphorylation (Lim et al., 2019).

Further detailed discussion regarding the role of autophagy specifically in the kidney diseases can be found in recent review articles (Tang et al., 2020; Zheng et al., 2020).

### Infection

Autophagy is also important antimicrobial defense mechanism against diverse infectious agents such as *Mycobacterium tuberculosis, Salmonella or Streptococcus*. Autophagy can contain cytosolic bacteria escaping from endosome/vacuole and induce maturation of phagosome into phagolysosome (Gutierrez et al., 2004; Nakagawa et al., 2004; Wild et al., 2008). In the intestine, secretory autophagy from Paneth cells mediates release of lysozyme, a critical antimicrobial peptide, and is an important host response against bacterial infection, which is defective in patients with Crohn’s disease (Bel et al., 2017).

In a paper studying the effect of autophagy enhancer on infection, Tat-beclin 1 reduced replication of *Listeria monocytogenes* and viruses such as *Sindbis* virus, chikungunya virus, West Nile virus and HIV *in vitro* and improved clinical outcome of such infections *in vivo* (Shoji-Kawata et al., 2013).

A77 1726, an active metabolite of anti-inflammatory drug leflunomide, has been reported to restrict *Salmonella* growth through AMPK activation and autophagy enhancement (Zhuang et al., 2020).

SMER 28 has also been reported to have capability to kill *Mycobacterium* (Floto et al., 2008).

Flubendazole, an autophagy enhancer identified in a screening using GFP-RFP-LC3 system and a chemical library, inhibited HIV transfer from dendritic cells to T cells and promoted clearance of intracellular bacteria (Chauhan et al., 2015).

Everolimus or rapamycin has also been reported to suppress productive infection of HIV (Cloherty et al., 2021).

Intriguingly, therapeutic effect of a novel TFEB activator against COVID-19 was reported in a preprint paper. In corona virus infection, TFEB was shown to be degraded through proteasomal degradation. BC18630 selected by an *in silico* screening of inhibitors against DCAF7, a putative E3 ligase mediating degradation of TFEB, was shown to reduce viral load and lung injury in a Syrian hamster model of SARS-CoV-2 infection (Liu et al., 2021). Modulators of autophagy, lysosome or TFEB could have therapeutic effects against COVID-19 pandemic caused by infection with SARS-CoV-2 that egresses host cells through lysosomal trafficking and disrupts lysosomal function (Ghosh et al., 2020).

In contrast to the protective role of autophagy against infectious diseases, some pathogens such as *H. pylori* or uropathogenic *E. coli* have been reported to hijack host autophagic machinery for their survival and growth (Huab et al., 2020). In this case, inhibition of autophagy may have a therapeutic effect.

### Lysosomal Storage Diseases

LSDs are a group of rare diseases in which lysosomal function is primarily impaired due to genetic causes and characterized by accumulation of excessive substrates in lysosome, leading to lysosomal dysfunction and a variety of systemic manifestations including neurodegeneration (Darios and Stevanin, 2020). As lysosomal dysfunction and autophagy impairment are observed in most of the LSDs, enhancement of autophagic or lysosomal activity could be a new modality to treat such diseases.

2-hydroxypropyl-β-cyclodextrin, an excipient and cholesterol-extracting agent, has been shown to activate TFEB and accelerate clearance of proteolipid aggregates in cells from patients with neuronal ceroid lipofuscinosis, a LSD (Song et al., 2014).

Trehalose has also been reported to attenuate inflammation of the brain and retina and to improve vision by activating TFEB and autophagic activity in a mouse model of mucopolysaccharidosis IIIB (MPS IIIB), a LSD caused by mutation of α-*N*-acetylglucosaminidase (NAGLU) (Lotfi et al., 2018).

### Ocular Diseases

The risk of eye diseases such as macular degeneration is exponentially increasing in advanced age (Luu and Palczewski, 2018), which might be related to dysregulated autophagy in aging (Kivinen, 2018).

Rosiglitazone has also been reported to attenuate post-operative fibrosis in an animal model of glaucoma, which was associated with autophagy enhancement (Zhang F. et al., 2019).

Artesunate (ART) is a semi-synthetic derivative of aforementioned artemisinin, has also been reported to ameliorate diabetic retinopathy through AMPK/SIRT1 activation (Li et al., 2021).

ML-SA1, aforementioned agonist of lysosomal TRPML1 Ca^2+^ channel, has been shown to protect retinal structure, reduce apoptosis of photoreceptor cells and improve vision-dependent behavior in a rat model of retinal detachment, which was accompanied by enhanced autophagy (Yan et al., 2021).

Apigenin, a well-known antioxidant and anti-inflammatory flavonoid, has been reported to improve clinical index and reduce intraocular oxidative damage in a mouse model of age-related macular degeneration (AMD) by upregulating autophagy and expression of *Nrf2*, a master regulator of anti-oxidative gene expression (Zhang et al., 2020c).

Effects of Rapamycin in an animal model of endotoxin-induced uveitis and retinitis model have also been studied, which demonstrated suppression photoreceptor degeneration and inflammation together with inhibition of NF-κB or mTOR and enhancement of autophagy (Okamoto et al., 2016).

## Autophagy Inhibitor

Inhibitors of autophagy have been developed mostly for application in cancer. Autophagy has been reported to inhibit or accelerate the development of cancer, depending on the stage of carcinogenesis as summarized in the review (White, 2012). Thus, autophagy deficiency could promote the initiation of cancer by producing reactive oxygen species and inducing chromosomal abnormality. In a similar vein, cell death due to autophagy hyperactivation or autophagic cell death has been reported to limit chromosomal instability during replicative crisis causing telomeric DNA damage (Nassour et al., 2019). Some autophagy genes act as tumor suppressor genes (Qu et al., 2003; Liang et al., 2006; Cianfanelli et al., 2015). At the later stage of cancer development, autophagy can accelerate the progression of cancer by supplying amino acids or energy that is necessary for the progression of cancer through tumor cell-autonomous or non-tumor cell-autonomous manner (Kimmel and White, 2017). Autophagy of host cells might play a role in the maintenance of serum arginine level that is necessary for tumor growth (Poillet-Perez et al., 2021).

Several autophagy inhibitors have been developed as potential anti-cancer drugs such as chloroquine or ULK1 inhibitors (Chude and Amaravadi, 2017; Martin et al., 2018), exploiting metabolic requirement of cancer cells. For instance, chloroquine in conjunction with leucine-free diet has been reported to suppress growth of melanoma that is resistant to mTORC1 inhibition and autophagy activation by leucine deprivation (Sheen et al., 2011). In contrast, the effect of ULK1/2 inhibition has been questioned since abrogation of ULK1/2-mediated autophagy could not block extracellular protein-dependent cancer cell growth (Palm et al., 2015). Further discussion regarding the role of autophagy in the pathogenesis of cancer or that of autophagy modulators in the cancer treatment is beyond the scope of this article, and the readers are encouraged to consult excellent review papers (White, 2012; Amaravadi et al., 2017; Levy et al., 2017; Miller and Thorburn, 2021).

## New Approaches for Autophagic or Lysosomal Degradation of Selective Targets

Recently, novel autophagic or lysosomal protein degradation techniques such as lysosome targeting chimera (LYTAC), autophagy-targeting chimera (AUTAC) or autophagy-tethering compound (ATTEC) have been developed (Ding et al., 2021). LYTAC comprises an antibody fused to mannose-6-phosphate (M6P) that can be recognized by cation-independent M6P receptor (CI-M6PR), a lysosomal trafficking receptor (Banik et al., 2020). Using this method, extracellular targets such as apolipoprotein E (APOE) or plasma membrane-bound targets such as epidermal growth factor receptor (EGFR) could be targeted to endosome or lysosome for degradation. AUTAC consists of warheads to selective targets, linker and guanine degradation tag inducing S-guanylation-mediated autophagic degradation (Takahashi et al., 2019). This technique may allow degradation of large molecules, protein aggregates, organelles such as mitochondria or bacteria which has not been possible with PROTAC (proteolysis-targeting chimera) utilizing ubiquitin E3 ligase-binding ligands (Ding et al., 2021). ATTEC employs compounds interacting with both mutant huntingtin (HTT) protein targets and LC3, directing target proteins to autophagic degradation without ubiquitination (Li et al., 2019). These novel approaches will provide outstanding tools for the development of next-generation modulators of autophagy or lysosomal degradation.

## Conclusion and Future Perspectives

While the significance of autophagy in the physiological function and pathogenesis of diverse diseases or aging is becoming more and more clear, authentic pharmacological autophagy modulators are still not available in the clinics except drugs that have already been used but were found to have autophagy-modulating effects later. Pharmacological modulation of specific processes or molecules among a wide array of the related genes or proteins at the specific sites is still not possible, precluding the use of autophagy modulators in the current circumstances. Lack of reliable biomarkers and suitable assay systems for measurement of autophagic flux in humans is also a big hurdle. Nonetheless, as detailed molecular mechanisms of selective autophagy are being discovered, specific modulation of specialized aspects of autophagy possibly at the specific tissues or sites will not be a remote possibility, which will be of a paramount importance for clinical application of autophagy modulators.

However, some caution might be exercised in the use of autophagy enhancer for treatment of human diseases. Lysosomal dysfunction is frequently observed in diverse tissues of patients with Alzheimer’s disease, obesity, T2D and associated conditions or aging (Hütter et al., 2007; Liu W. J. et al., 2015; Bordi et al., 2016; Zhao et al., 2019; Fernando et al., 2020; Kim et al., 2021a). In such conditions, accumulation of autophagic intermediates due to lysosomal dysfunction may be further increased after administration of autophagy enhancers activating earlier steps of autophagy, which might lead to autophagic stress or autophagic cell death (Zheng et al., 2020). Thus, it might be necessary to manage lysosomal dysfunction together with or before administration of autophagy enhancers. Possibility of aggravation of pre-existing cancer or infection due to pathogens exploiting host autophagic machinery for their survival by autophagy enhancers should also be kept in mind. Therefore, maintaining the exquisite balance of timing, duration and the extent of autophagy modulation depending on the types and stages of the diseases is crucial.
